# Giant Mpemba Effect via Weak Interactions in Open Quantum Systems

**DOI:** 10.3390/e28040427

**Published:** 2026-04-10

**Authors:** Stefano Longhi

**Affiliations:** 1 Dipartimento di Fisica, Politecnico di Milano, Piazza L. da Vinci 32, I-20133 Milano, Italy; stefano.longhi@polimi.it; 2 IFISC (UIB-CSIC), Instituto de Física Interdisciplinar y Sistemas Complejos, Campus Universitat de les Illes Balears, E-07122 Palma de Mallorca, Spain

**Keywords:** quantum Mpemba effect, open quantum systems, relaxation dynamics, metastability

## Abstract

The Mpemba effect refers to the counterintuitive situation in which a system initially farther from equilibrium can relax faster than one that starts closer to it. In quantum systems, the effect is enriched by the presence of coherent dynamics, dissipation, and metastable manifolds associated with long-lived Liouvillian modes. Here we demonstrate a giant Mpemba effect in open quantum systems, where relaxation can be either hyper-accelerated or dramatically slowed depending on the initial state. We focus on weakly-coupled particle-conserving bosonic networks, each of which independently relaxes rapidly to a unique stationary state. When a weak coherent interaction is introduced, the composite system typically develops slow metastable modes and a hierarchy of relaxation timescales. We show that by tailoring the interaction Hamiltonian, these slow modes can be effectively suppressed for a broad class of initial states satisfying a minimal global requirement, enabling ultrafast relaxation even when the system starts far from equilibrium. Conversely, other initial states—sometimes arbitrarily close to the stationary state—may remain trapped in the metastable manifold and decay anomalously slowly. This mechanism provides a general route to engineer giant Mpemba effects, offering new possibilities for controlling dissipative dynamics, accelerating state preparation, and manipulating relaxation processes in complex quantum devices.

## 1. Introduction

The Mpemba effect [1,2,3], originally reported in classical settings, refers to the counterintuitive phenomenon whereby a system initially farther from equilibrium relaxes faster than one initially closer to it; while the earliest discussions were framed around the anomalous cooling of water [4,5,6], it is now recognized that the effect arises in many types of nonequilibrium systems [1,2,7,8,9,10,11,12,13,14,15,16,17,18,19], including granular fluids, polymer networks, spin glasses, and stochastic processes. The idea of a “strong Mpemba effect” was first formalized in classical systems in Ref. [9], where it was shown that for carefully chosen initial conditions the relaxation time can jump to a much smaller value, leading to exponentially faster equilibration. Subsequent research has extended these concepts to quantum systems [3,20], focusing on either the unitary dynamics of *closed* quantum systems [21,22,23,24,25,26,27,28,29,30,31,32,33,34,35,36,37] or the dissipative evolution in *open* quantum systems [20,38,39,40,41,42,43,44,45,46,47,48,49,50,51,52,53,54,55,56,57,58,59,60,61,62,63,64,65,66,67,68,69,70,71,72,73,74,75,76,77,78,79,80], with interesting generalizations such as the Pontus–Mpemba effect [81,82,83,84,85,86]. For recent reviews see [2,3]. In the present work we focus exclusively on open quantum systems, where the combined action of coherent Hamiltonian evolution and coupling to an environment gives rise to rich relaxation structures governed by the spectrum of the Liouvillian superoperator.

Open quantum systems can exhibit metastability and a hierarchy of relaxation timescales [41,87,88,89,90,91,92,93,94]. Fast-decaying modes dominate the early evolution, while slowly decaying modes—often associated with metastable manifolds or nearly conserved quantities—control the long-time approach to stationarity. This separation of timescales creates the conditions under which the quantum Mpemba effect may appear [41]: if an initial state has negligible overlap with the slowest-decaying modes, it may relax much faster than a state that is initially closer to the stationary state but has significant projection onto the metastable subspace. The role of metastability in realizing the Mpemba effect is well-known for classical systems as well [19,95,96,97]. The *giant* Mpemba effect refers to an extreme form of anomalous relaxation in which certain initial states undergo *hyper-accelerated* relaxation by avoiding all long-lived metastable modes. In this regime, a state that is initially very far from equilibrium can relax *orders of magnitude faster* than another state much closer to equilibrium—hence the term *giant*. However, observing a giant Mpemba effect is typically challenging. The special initial states that avoid exciting any slow mode are generally highly model-dependent and depend sensitively on the spectral structure of the Liouvillian. As a result, they are rarely known a priori, and their preparation can be difficult or even experimentally inaccessible. Furthermore, even slight deviations from precise initial state preparation can exponentially reduce speed-up relaxation to stationarity [98]. Transient stochastic resetting, where during an initial time interval of finite duration the dynamics are interrupted by resets that take the system to a designated state at randomly selected times, has been recently suggested to avoid fine tuning of the initial state [78].

In this work, we propose a conceptually different strategy to achieve a giant Mpemba effect in open quantum systems via the choice of interaction Hamiltonians, providing a complementary approach to the initial-state tuning approach [7,20]. Instead of relying on unusual or finely tuned initial states, we demonstrate that one can tailor the interaction Hamiltonian between two sub-systems so as to *guarantee* that a broad and physically natural class of initial states automatically avoids exciting the entire metastable manifold. This provides a robust and experimentally feasible route to hyper-accelerated relaxation, one that does not require detailed knowledge of the slow modes themselves. For definiteness, we consider two weakly coupled particle-conserving bosonic networks, labeled *A* and *B*, a main illustrative case. The analysis, however, could be extended to a larger number of weakly-coupled subsystems and beyond bosonic models. Each subsystem is governed by its own particle-conserving Hamiltonian and is independently subject to particle-conserving dissipators, such as local or global dephasing, incoherent hopping, or mode-mixing channels. In the absence of interactions, each subsystem relaxes to a unique stationary state on a fast timescale, with no long-lived or metastable components. When a weak coherent interaction $\epsilon H_{I}$ is introduced between the networks, new dynamical features emerge: the coupling typically generates slow Liouvillian modes whose decay rates scale with the small parameter ϵ as $∼\epsilon^{2}$, producing a hierarchy of timescales absent in the uncoupled dynamics. This slow manifold is responsible for metastability in the full system and controls the long-time relaxation. However, for tailored interaction Hamiltonians $H_{I}$, the system can be readily prepared in such a way that the slow modes are never excited. In this case, the dynamics remain confined to the fast subspace, and the approach to the stationary state is hyper-accelerated despite the presence of the weak coupling. This provides a natural realization of the giant Mpemba effect in a broad family of physically relevant open quantum systems.

Our results show that weak interactions combined with particle-conserving dissipation naturally generate hierarchical relaxation timescales, and that a suitably designed interaction Hamiltonian can suppress the activation of slow metastable modes for a broad class of initial states. This mechanism enables giant Mpemba effects without requiring finely tuned or model-specific state preparation, underscoring their broad relevance as a tool for controlling relaxation in complex quantum devices. The framework developed here demonstrates how ultra-fast relaxation can be systematically induced in composite quantum systems, with potential applications in quantum simulators, reservoir engineering, and state-reset protocols.

## 2. Model, Relaxation Dynamics and Giant Mpemba Effect

### 2.1. Model

We investigate open quantum systems exhibiting hierarchical relaxation and metastability, realized as a set of weakly coupled *M* dissipative subsystems, typically representing *M* bosonic networks. We introduce a weak interaction (coupling) term $\epsilon H_{I}$ among the *M* subsystems, corresponding to weak hopping links between sites belonging to different subsystems, each with characteristic rate ϵ. The coupling strength ϵ is assumed to be much smaller than the intrinsic relaxation rate *g* of the non-interacting subsystems, so that the interaction acts only as a perturbation. For definiteness, we begin by analyzing the case of two subsystems, *A* and *B* (M=2), although the discussion extends *mutatis mutandis* to a larger number *M* of weakly coupled subsystems. As a concrete example, we take *A* and *B* to be bosonic dissipative networks composed of $L_{A}$ and $L_{B}$ sites, respectively, with local Hamiltonians $H_{A}$ and $H_{B}$. The two subsystems interact via a coherent coupling $\epsilon H_{I}$ and typically with $L_{A}\gg L_{B}$. The full Hamiltonian of the composite system reads

(1) $$H=H_{A}⊗I_{B}+I_{A}⊗H_{B}+\epsilon H_{I},$$

where $I_{A}$ and $I_{B}$ denote the identity operators on the Hilbert spaces of *A* and *B*. The interaction $\epsilon H_{I}$ mediates coherent coupling between the networks and may generate correlations or entanglement, while the small parameter ϵ/g≪1 ensures that the coupling acts as a perturbation on the otherwise independent dissipative dynamics. The evolution of the full density operator ρ(t) is governed by the Gorini–Kossakowski–Sudarshan–Lindblad master equation

(2) $$\frac{d\rho }{dt}=-i\left[H,\rho \right]+\sum_{\mu }D\;\left[L_{\mu }^{A}\right]\rho +\sum_{\mu }D\;\left[L_{\mu }^{B}\right]\rho ,$$

where the dissipators act separately on each subsystem. Here,

(3) $$D\left[L\right]\rho =L\rho L^{†}-\frac{1}{2}\left\{L^{†}L,\rho \right\},$$

and $\left\{L_{\mu }^{A}\right\}$ and $\left\{L_{\mu }^{B}\right\}$ represent the dissipative channels in subsystems *A* and *B*, respectively.

We focus on particle-conserving Hamiltonians and dissipators which preserve the total number of excitations and thereby enforce an excitation-number hierarchy dictated by the initial state. Formally, this requires $[H_{A},N_{A}]=0$, $[H_{B},N_{B}]=0$, $[H_{I},N]=0$, and $\left[N_{A},L_{\mu }^{A}\right]=\left[N_{B},L_{\mu }^{B}\right]=0$ for all μ, where $N_{A}=\sum_{k=1}^{L_{A}}a_{k}^{†}a_{k}$, $N_{B}=\sum_{k=1}^{L_{B}}b_{k}^{†}b_{k}$ and $N=N_{A}+N_{B}$ are the particle-number operators, $a_{k}^{†}$ and $a_{k}$ ($k=1,\ldots ,L_{A}$) are the bosonic creation and annihilation operators of subsystem *A*, and $b_{k}^{†}$ and $b_{k}$ ($k=1,\ldots ,L_{B}$) are those of subsystem *B*. For example, linear bosonic networks described by the quadratic Hamiltonians $H_{A}=\sum_{i,j=1}^{L_{A}}h_{i,j}^{\left(A\right)}a_{i}^{†}a_{j}$ and $H_{B}=\sum_{i,j=1}^{L_{B}}h_{i,j}^{\left(B\right)}b_{i}^{†}b_{j}$ do conserve the total number of excitations. Explicit examples of particle-number-conserving dissipators include local dephasing, e.g., $L_{k}^{A}=a_{k}^{†}a_{k}$; incoherent hopping between sites, $L_{kl}^{A}=a_{k}^{†}a_{l}$; and general mode-mixing processes, $L_{\alpha }^{A}=\sum_{k\neq l}c_{\alpha kl}a_{k}^{†}a_{l}$ with complex amplitudes $c_{\alpha kl}$, which redistribute excitations among multiple modes while preserving total particle number. Analogous expressions hold for subsystem *B*.

### 2.2. Relaxation Dynamics

#### 2.2.1. Non-Interacting Limit

In the limit of vanishing interaction (ϵ=0), the full Liouvillian L of the system factorizes additively as $L=L_{0}=L_{A}⊗I_{B}+I_{A}⊗L_{B}$, where $L_{A}$ and $L_{B}$ are the Liouvillians of subsystems *A* and *B*, respectively, and $I_{A}$ and $I_{B}$ denote the identity superoperators on the operator spaces of *A* and *B*. In this case, the subsystems evolve independently. We assume that they relax toward unique non-equilibrium stationary states, denoted $\rho_{A}^{\left(N_{A}\right)}$ and $\rho_{B}^{\left(N_{B}\right)}$, within the specified excitation sectors $N_{A}$ and $N_{B}$ of their Hilbert spaces. We assume that each subsystem does not host long-lived metastable states, so that relaxation toward stationarity is fast and occurs on the timescale $\tau_{f}∼1/g$, set by the inverse of the spectral gaps *g* of $L_{A}$ and $L_{B}$, in the absence of non-normal effects [61,99,100]. Within the sector of Hilbert space with fixed particle number *N*, the zero eigenvalue of $L_{0}$ corresponding to stationary states is clearly (N+1) degenerate, since any state $\rho_{A}^{\left(n\right)}⊗\rho_{B}^{\left(N-n\right)}$, with n=0,1,2,…,N, is an eigenvector of $L_{0}$ with zero eigenvalue. As shown in Appendix A and Section 2.2.2, when subsystems A and B are weakly coupled via the Hamiltonian term $\epsilon H_{I}$ the degeneracy is lifted and such eigenstates are mixed, leading to the appearance of *N* long-lived (metastable) eigenstates of the Liouvillian L and a unique stationary state $\rho_{E}^{\prime }$.

Let us then consider the broad class of initial states in the form of the mixture

(4) $$\rho \left(0\right)=p_{A}\;\rho_{A}\left(0\right)⊗{|0\rangle }_{B}\langle 0|+p_{B}{\;|0\rangle }_{A}\langle 0|⊗\rho_{B}\left(0\right),$$

where $\rho_{A}\left(0\right)$ and $\rho_{B}\left(0\right)$ are the initial reduced density operators of subsystems *A* and *B*, belonging to the $N_{A}=N_{B}\equiv N$ excitation manifolds, *N* is the total number of particles in the system, $p_{A}+p_{B}=1$, and ${|0\rangle }_{A}$, ${|0\rangle }_{B}$ are the vacuum states of the respective subsystems. Such an initial state rapidly relaxes toward the non-equilibrium stationary state

(5) $$\rho_{E}=p_{A}\;\rho_{A}^{\left(N\right)}⊗{|0\rangle }_{B}\langle 0|+p_{B}{\;|0\rangle }_{A}\langle 0|⊗\rho_{B}^{\left(N\right)}$$

on the fast timescale $\tau_{f}$, determined by the spectral gaps of the independent Liouvillians $L_{A}$ and $L_{B}$.

#### 2.2.2. Weakly Interacting Regime

When the weak interaction $\epsilon H_{I}$ is switched on, within the sector of Hilbert space with fixed particle number *N*, the degeneracy of the zero eigenvalue of the Liouvillian is lifted and, as shown in Appendix A, the perturbation mixes the (N+1) eigenvectors $\rho_{A}^{\left(n\right)}⊗\rho_{B}^{\left(N-n\right)}$, yielding a unique stationary state $\rho_{E}^{\prime }$ and *N* metastable (long-lived) eigenstates of the Liouvillian L (slow manifold modes). The non-equilibrium stationary state $\rho_{E}^{\prime }$ of the full system generally differs from the non-interacting stationary state $\rho_{E}$ of Equation (5). In particular, since $\rho_{E}$ will in general not commute with $\epsilon H_{I}$, its projection onto the slow-eigenmode manifold of the full Liouvillian L contains long-lived components. The particle-number conservation laws in each subsystem are weakly broken by the small coherent perturbation $\epsilon H_{I}$, and the dynamics exhibit a clear separation of timescales provided that ϵ≪g. In this limit, a perturbative analysis using a standard P−Q projection operator formalism (Nakajima–Zwanzig type) can be performed, which systematically separates the dynamics into a slow subspace associated with the stationary states of the uncoupled subsystems (slow manifold) and a fast subspace corresponding to the relaxation within the individual subsystems. Initially, the system undergoes a fast relaxation toward the subspace indexed by the nearly conserved particle numbers, similar to what occurs in weakly dissipative integrable models [36,101]. This fast decay, occurring on timescale $\tau_{f}$, drives each subsystem to its local stationary states, $\rho_{A}^{\left(n\right)}$ and $\rho_{B}^{\left(N-n\right)}$. Subsequently, a much slower evolution toward the true stationary state $\rho_{E}^{\prime }$ of the full Liouvillian with the interacting term takes place on a timescale $\tau_{s}∼{\left(g/\epsilon \right)}^{2}$. More precisely (see Appendix A), the solution of Equation (2) can be written as

$$\rho \left(t\right)=\sum_{n=0}^{N}p_{n}\left(t\right)\;\rho_{A}^{\left(n\right)}⊗\rho_{B}^{\left(N-n\right)}\;+\;O\left(\epsilon /g\right),$$

where the populations $p_{n}\left(t\right)$ evolve on the slow timescale $\tau_{s}$, and the O(ϵ) correction is a rapidly decaying component lying outside the slow manifold. Appendix A provides the detailed derivation of the slow evolution of populations $p_{n}\left(t\right)$, which is effectively described by classical rate equations [Equation (A16)], with transition rates of order ${\left(\epsilon /g\right)}^{2}$ between the product equilibrium states. Hence, the physical picture of the slow dynamics appears to be a form of “incoherent hopping” or diffusive transport across the weak link $\epsilon H_{I}$.

**Figure 1:**
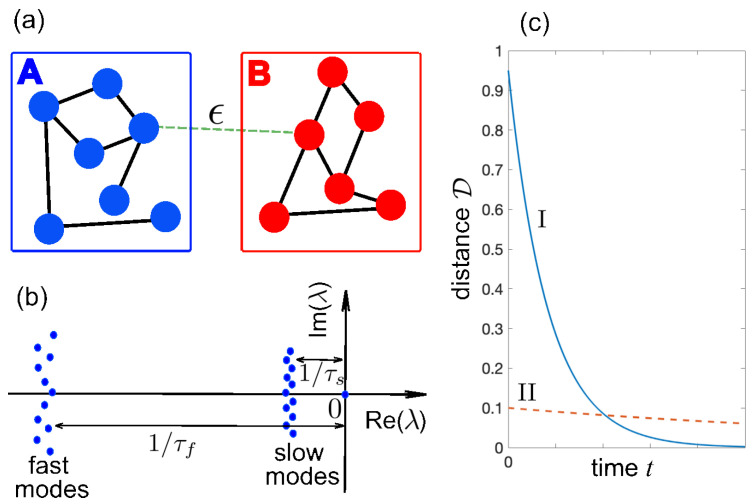
(**a**) Schematic of two dissipative quantum subsystems *A* and *B* weakly coupled via a coherent Hamiltonian $\epsilon H_{I}$. The two subsystems can be, for example, particle-number-conserving dissipative bosonic networks. (**b**) Typical eigenvalue spectrum of the Liouvillian L of the full system for weak interaction in a given excitation number sector of Hilbert space. The zero eigenvalue corresponds to the stationary state $\rho_{E}^{\prime }$, whereas the other eigenvalues form two manifolds of fast decaying modes, with large decay rates $∼1/\tau_{f}$, and slow decaying modes (metastable states) with small decay rates vanishing as ϵ→0. (**c**) Schematic of the giant Mpemba effect. Initial state I, which is very far from the equilibrium state, does not excite the slow modes and rapidly relaxes toward $\rho_{E}^{\prime }$ on the fast timescale $\tau_{f}$. Conversely, state II, which is very close to the equilibrium state, excites the slow modes and thus relaxation toward $\rho_{E}^{\prime }$ is very slow and occurs on the timescale $\tau_{s}$, diverging as ϵ→0.

For small but finite ϵ/g, the hierarchy of fast and slow temporal dynamics is reflected in the spectrum of the full Liouvillian L: fast-decaying modes correspond to the eigenvalues inherited from the unperturbed Liouvillians $L_{A}$ and $L_{B}$, while a set of slow modes is generated by the weak interaction [Figure 1b]. These slow modes encode the dynamics of the populations $p_{n}\left(t\right)$, described by a rate equation model. As shown in Appendix A, there are N+1 populations but exactly *N* slow-decaying modes (the remaining one corresponds to the stationary eigenvector with eigenvalue zero), where *N* is the total number of excitations in the fixed particle-number sector of the dynamics established by the initial condition. Denoting by $r_{\alpha }^{\left(f\right)}$, $l_{\alpha }^{\left(f\right)}$ and $r_{\beta }^{\left(s\right)}$, $l_{\beta }^{\left(s\right)}$ the right and left eigenvectors of L in the fast and slow manifolds, with $Lr_{\alpha }^{\left(f\right)}=\lambda_{\alpha }^{\left(f\right)}r_{\alpha }^{\left(f\right)}$ and $Lr_{\beta }^{\left(s\right)}=\lambda_{\beta }^{\left(s\right)}r_{\beta }^{\left(s\right)}$, and decay rates satisfying Re(λα(f))∼−1/τf≪Re(λβ(s))∼−1/τs<0, the relaxation dynamics is

(6) $$\rho \left(t\right)=\rho_{E}^{\prime }+\sum_{\alpha }C_{\alpha }^{\left(f\right)}r_{\alpha }^{\left(f\right)}\exp\left(\lambda_{\alpha }^{\left(f\right)}t\right)+\sum_{\beta }C_{\beta }^{\left(s\right)}r_{\beta }^{\left(s\right)}\exp\left(\lambda_{\beta }^{\left(s\right)}t\right),$$

with spectral amplitudes $C_{\alpha }^{\left(f\right)}=\mathrm{Tr}\left(l_{\alpha }^{(f)†}\rho \left(0\right)\right)$ and $C_{\beta }^{\left(s\right)}=\mathrm{Tr}\left(l_{\beta }^{(s)†}\rho \left(0\right)\right)$. Hyper-accelerated relaxation requires initial states ρ(0) with zero overlap with all slow modes, i.e., $C_{\beta }^{\left(s\right)}=0$ for all β. Such states are highly model-dependent and rarely known a priori, making their preparation challenging or experimentally inaccessible.

An alternative approach is to tailor the interaction Hamiltonian $\epsilon H_{I}$ so that the slow modes are automatically unexcited. For specific forms of $\epsilon H_{I}$ and appropriate initial conditions (typically determined by degeneracies of the non-interacting stationary states), one can achieve $[H_{I},\rho_{E}]=0$. In this case, $\rho_{E}$ coincides with the stationary state $\rho_{E}^{\prime }$ of the interacting system, and the slow modes are not excited: relaxation then occurs entirely on the fast timescale $\tau_{f}$.

One can design several particle-conserving interaction Hamiltonians that satisfy the condition $[H_{I},\rho_{E}]=0$. For example, let ${|a\rangle }_{A}$ and ${|b\rangle }_{B}$ be right eigenvectors of $\rho_{A}^{\left(N_{A}\right)}$ and $\rho_{B}^{\left(N_{B}\right)}$ with nonzero real eigenvalues $\lambda_{a}$ and $\lambda_{b}$, and let us consider the particle-conserving interaction Hamiltonian

(7) $$\epsilon H_{I}=\epsilon \left({|a\rangle }_{A}{\langle 0|}_{A}⊗{|0\rangle }_{B}{\langle b|}_{B}+H.c.\right).$$

By direct calculation, it turns out that $[H_{I},\rho_{E}]=0$ if and only if

(8) $$p_{A}\lambda_{a}=p_{B}\lambda_{b}.$$

In fact, since $\rho_{E}=p_{A}\;\rho_{A}^{\left(N_{A}\right)}⊗{|0\rangle }_{B}{\langle 0|}_{B}+p_{B}{\;|0\rangle }_{A}{\langle 0|}_{A}⊗\rho_{B}^{\left(N_{B}\right)}$, taking into account that ${\langle 0|}_{B}{|b\rangle }_{B}={\langle 0|}_{A}{|a\rangle }_{A}=0$, one directly computes

$$H_{I}\rho_{E}=p_{A}\lambda_{a}{\;|0\rangle }_{A}{\langle a|}_{A}⊗{|b\rangle }_{B}{\langle 0|}_{B}+p_{B}\lambda_{b}{\;|a\rangle }_{A}{\langle 0|}_{A}⊗{|0\rangle }_{B}{\langle b|}_{B}$$

and

$$\rho_{E}H_{I}=p_{B}\lambda_{b}{\;|0\rangle }_{A}{\langle a|}_{A}⊗{|b\rangle }_{B}{\langle 0|}_{B}+p_{A}\lambda_{a}{\;|a\rangle }_{A}{\langle 0|}_{A}⊗{|0\rangle }_{B}{\langle b|}_{B},$$

where we used $\rho_{A}^{\left(N_{A}\right)}{|a\rangle }_{A}=\lambda_{a}{|a\rangle }_{A}$ and $\rho_{B}^{\left(N_{B}\right)}{|b\rangle }_{B}=\lambda_{b}{|b\rangle }_{B}$. Thus, the commutator takes the simple form

$$\left[H_{I},\rho_{E}\right]=\left(p_{A}\lambda_{a}-p_{B}\lambda_{b}\right)\;\left({|0\rangle }_{A}{\langle a|}_{A}⊗{|b\rangle }_{B}{\langle 0|}_{B}-H.c.\right).$$

This means that, since $L_{0}\rho_{E}=0$ and $L=L_{0}-i\epsilon \left[H_{I},\cdot \right]$, $\rho_{E}$ is a fixed point of the full Liouvillian L if and only if $p_{A}\lambda_{a}=p_{B}\lambda_{b}$. This proves why the interaction term does not modify the stationary state under the matching condition (8). As a consequence, any initial state ρ(0) given by Equation (4) with probabilities $p_{A}$ and $p_{B}$ satisfying the mathcing condition (8) does not excite slow eigenvectors and thus relaxes toward the stationary state on the fast timescale $\tau_{f}$, regardless of specific forms of $\rho_{A}\left(0\right)$ and $\rho_{B}\left(0\right)$. Conversely, initial states of the form (4) with probabilities $p_{A}$ and $p_{B}$ that do not satisfy the condition (8) display two different relaxation timescales: a fast relaxation on a timescale of order $∼\tau_{f}$ that brings the initial state to the metastable state $\rho_{E}$, and a slow relaxation on a timescale of order $∼\tau_{s}$ that brings the metastable state $\rho_{E}$ toward the stationary state $\rho_{E}^{\prime }$ of the interacting system.

A particularly simple and insightful case is the one where the stationary states in each subsystem A and B are maximally mixed states $\rho_{A}^{\left(N_{A}\right)}=\left(1/d_{A}\right)I_{A}$ and $\rho_{B}^{\left(N_{B}\right)}=\left(1/d_{B}\right)I_{B}$, where $d_{A}$ and $d_{B}$ are the dimensions of the Hilbert spaces in A and B within the $N_{A}$ and $N_{B}$ particle sectors, respectively. This situation is generally observed when dissipators describe global dephasing and/or incoherent hopping among modes of the networks. In this case, it can be readily shown that, provided that the condition

(9) $$\frac{p_{A}}{d_{A}}=\frac{p_{B}}{d_{B}}$$

is satisfied, the stationary state $\rho_{E}$ given by Equation (5) is the identity operator on its support, and therefore *any* particle-number-conserving Hamiltonian $\epsilon H_{I}$ commutes with $\rho_{E}$, and thus does not excite slow modes.

### 2.3. Giant Mpemba Effect

The giant Mpemba effect arises as follows. Assume that the Hilbert space of $L_{A}$ in the *N*-excitation sector is much larger than that of $L_{B}$ in the *N*-excitation sector. For example, when $N_{A}=N_{B}=1$, this occurs if the number of network nodes satisfies $L_{A}\gg L_{B}$. In this regime, for generic weak interactions $\epsilon H_{I}$, the distance $D(\rho_{E},\rho_{E}^{\prime })$ between the non-interacting and interacting stationary states is small, since subsystem *A* dominates the relaxation dynamics of the composite system. For the distance $D(\rho_{1},\rho_{2})$ between two density matrices, we adopt the trace distance (see, e.g., Ref. [3])

(10) $$D_{tr}\left(\rho_{1},\rho_{2}\right)=\frac{1}{2}\mathrm{Tr}\;\left|\rho_{1}-\rho_{2}\right|,$$

where $\left|X\right|=\sqrt{X^{†}X}$. To confirm that the effect is independent of the chosen distance measure, we additionally employ the Hilbert–Schmidt (or Frobenius) distance [3], defined by

$$D_{HS}\left(\rho_{1},\rho_{2}\right)=\sqrt{\mathrm{Tr}\;\{{\left(\rho_{1}-\rho_{2}\right)}^{2}\}}.$$

Consider two initial states, I and II, of the form in Equation (4). Initial state I has probabilities $p_{A}$ and $p_{B}$ satisfying Equation (8), with $\rho_{A}\left(0\right)$ far from $\rho_{A}^{\left(N\right)}$. The distance $D(\rho^{\left(I\right)}\left(0\right),\rho_{E}^{\prime })$ is large, yet because $[H_{I},\rho_{E}]=0$, the slow modes are not excited, and relaxation occurs on the fast timescale $\tau_{f}$. Initial state II has probabilities $p_{A}$ and $p_{B}$ that do not satisfy Equation (8), with $\rho_{A}\left(0\right)$ very close (or even identical) to $\rho_{A}^{\left(N\right)}$. Here, $D(\rho^{\left(II\right)}\left(0\right),\rho_{E}^{\prime })$ is small, but slow modes are activated, so after an initial fast transient, relaxation proceeds on the slow timescale $\tau_{s}$. This striking contrast—namely, that a state initially farther from equilibrium can relax orders of magnitude ($∼{\left(g/\epsilon \right)}^{2}$) faster than one nearer to equilibrium—exemplifies the giant Mpemba effect, as schematically shown in Figure 1c.

## 3. Illustrative Example: Weakly Coupled Bosonic Networks with Local Dephasing

As an illustrative example, we consider two bosonic networks *A* and *B*, such as two tight-binding bosonic chains, with $L_{A}$ and $L_{B}$ nodes, subject to local dephasing in each node at the same rate Γ. The Hamiltonians of the two subsystems *A* and *B* are

(11) $$H_{A}=\sum_{i,j=1}^{L_{A}}h_{i,j}^{\left(A\right)}a_{i}^{†}a_{j},\;H_{B}=\sum_{i,j=1}^{L_{B}}h_{i,j}^{\left(B\right)}b_{i}^{†}b_{j},$$

whereas the dissipators describing local dephasing at the rate Γ read

(12) $$L_{\mu }^{A}=\sqrt{\Gamma }\;a_{\mu }^{†}a_{\mu },\;L_{\mu }^{B}=\sqrt{\Gamma }\;b_{\mu }^{†}b_{\mu }.$$

The interaction Hamiltonian is assumed to cross-couple nodes among the two subsystems, and its general form is thus

(13) $$\epsilon H_{I}=\sum_{i,k}\left(h_{i,k}a_{i}^{†}b_{k}+H.c.\right).$$

The cross-coupling terms basically connect the two networks, removing the disconneted configuration in the ϵ=0 limit. The simplest interaction Hamiltonian that connects the two networks A and B corresponds to a single coupling link between two nodes of the networks, for example, nodes i=j=1, i.e.,

(14) $$\epsilon H_{I}=\epsilon \left(a_{1}^{†}b_{1}+b_{1}^{†}a_{1}.\right)$$

Let us first consider the single-excitation sector $N_{A}=N_{B}=1$. After letting $c_{k}=a_{k}$ for $k=1,2,\ldots ,L_{A}$ and $c_{k}=b_{k-L_{A}}$ for $k=L_{A}+1,L_{A}+2,\ldots ,L_{A}+L_{B}$, in the single-excitation manifold, the Hilbert space of the full interacting system is described by the states $|k\rangle =c_{k}^{†}|0\rangle$, with k=1,2,…,L and $L=L_{A}+L_{B}$. Introducing the density-matrix elements $\rho_{n,m}\left(t\right)=\langle n|\rho \left(t\right)|m\rangle$, their evolution equation is readily obtained from Equation (2) and reads

(15) $$\frac{d\rho_{n,m}}{dt}=-i\sum_{l=1}^{L}H_{n,l}\rho_{l,m}+i\sum_{l=1}^{L}\rho_{n,l}H_{l,m}-\Gamma \left(1-\delta_{n,m}\right)\rho_{n,m}$$

where $H_{n,m}=\langle n|H|m\rangle$ represents the matrix elements of the full Hamiltonian $H=H_{A}+H_{B}+\epsilon H_{I}$. The initial state ρ(0) is assumed to be of the form given by (4) with N=1. Dephasing primarily suppresses coherences between different states, driving the system toward a diagonal density matrix with populations tending to equalize across the network nodes. For ϵ=0, the stationary state $\rho_{E}=p_{A}\rho_{A}^{\left(1\right)}⊗{|0\rangle }_{B}\langle 0|+p_{B}{\;|0\rangle }_{A}\langle 0|⊗\rho_{B}^{\left(1\right)}$ is reached after a fast transient relaxation, where

(16) $$\rho_{A}^{\left(1\right)}=\frac{1}{L_{A}}I_{A}\;\;,\;\rho_{B}^{\left(1\right)}=\frac{1}{L_{B}}I_{B}$$

and $I_{A,B}$ are the $L_{A,B}\times L_{A,B}$ identity matrices. On the other hand, when the interaction is present (ϵ≠0), the stationary state is given by

(17) $$\rho_{E}^{\prime }=\frac{1}{L}I=\frac{1}{L}I_{A}⊗{|0\rangle }_{B}\langle 0|+\frac{1}{L}{|0\rangle }_{A}\langle 0|⊗I_{B}$$

where I is the identity operator in the Hilbert space of the full system, i.e., the L×L identity matrix. Remarkably, this stationary state is *independent* of the specific interaction Hamiltonian $\epsilon H_{I}$, and a single connecting link [Equation (14)] is enough to realize this stationary state. The condition $\rho_{E}=\rho_{E}^{\prime }$, which guarantees that the slow modes are bypassed and the relaxation occurs on the fast timescale, is satisfied whenever

(18) $$\frac{p_{A}}{L_{A}}=\frac{p_{B}}{L_{B}}=\frac{1}{L}.$$

By direct evaluation of the commutator $\left[H_{I},\rho_{E}\right]$, with $H_{I}$ given in Equation (13), one readily verifies that Equation (18) is the necessary and sufficient condition for $[H_{I},\rho_{E}]=0$. This condition (18) is precisely a special case of the global condition (9) given in Section 2.2.2, with $d_{A}=L_{A}$ and $d_{B}=L_{B}$ for $N_{A}=N_{B}=1$.

To illustrate the emergence of a giant Mpemba effect, let us consider the regime $L_{A}\gg L_{B}$ and compare the relaxation dynamics of two initial states, I and II, of the form specified in Equation (4). *State I* has probabilities $p_{A}$ and $p_{B}$ satisfying Equation (18), with $\rho_{A}\left(0\right)=\left|1\rangle \langle 1\right|$ and $\rho_{B}\left(0\right)=\left|L\rangle \langle L\right|$. Although this state is very far from equilibrium (in fact in *A* the excitation is confined to the single site |1〉), it has essentially no overlap with the slow Liouvillian modes and therefore relaxes rapidly. *State II* is defined by $p_{A}=1$ and $p_{B}=0$, with $\rho_{A}\left(0\right)=\left(1/L_{A}\right)\sum_{k=1}^{L_{A}}\left|k\rangle \langle k\right|$. This state is much closer to the equilibrium state (17), yet it excites slow Liouvillian modes and thus relaxes extremely slowly, in stark contrast with state I.

**Figure 2:**
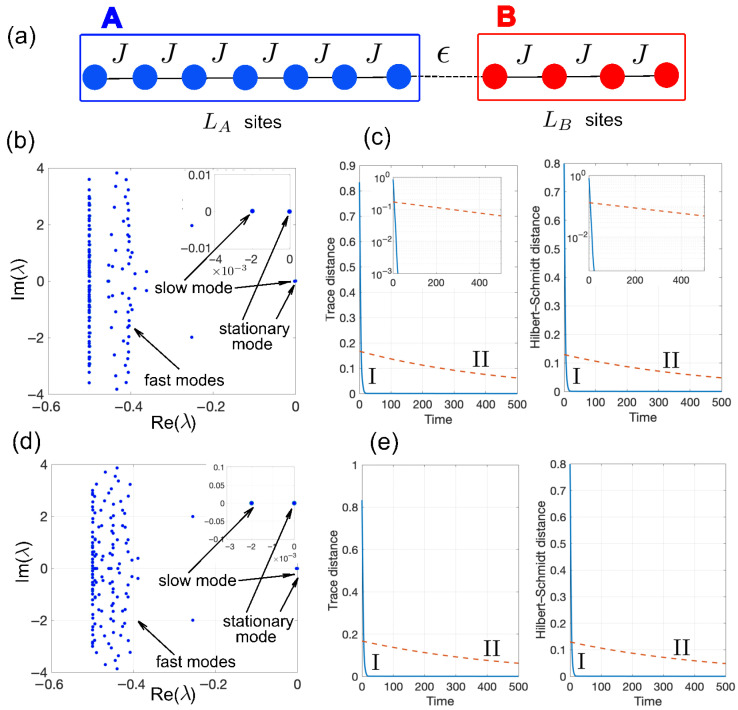
(**a**) Schematic of two dissipative bosonic chains *A* and *B* with nearest-neighbor hopping rate *J*, connected by a weak link of hopping strength ϵ. (**b**) Numerically computed eigenvalue spectrum λ of the Liouvillian L in the N=1 excitation sector for $L_{A}=10$, $L_{B}=2$, J=1, ϵ/J=0.05, and Γ/J=0.5. The inset shows a zoom of the region near λ=0, highlighting the unique zero eigenvalue corresponding to the stationary state $\rho_{E}^{\prime }$ and the nearby slow mode. (**c**) Trace distance $D_{tr}\left(\rho \left(t\right),\rho_{E}^{\prime }\right)$ (left panel) and Hilbert–Schmidt distance $D_{HS}\left(\rho \left(t\right),\rho_{E}^{\prime }\right)$ (right panel) for the relaxation dynamics of the two initial states I and II. State I is prepared as $\rho \left(0\right)=p_{A}\left|1\rangle \langle 1\right|+p_{B}\left|L\rangle \langle L\right|$ with $p_{A}=L_{A}/L=10/12$, $p_{B}=L_{B}/L=2/12$, $L=L_{A}+L_{B}=12$. State II is defined as $\rho \left(0\right)=\frac{1}{L_{A}}\sum_{k=1}^{L_{A}}\left|k\rangle \langle k\right|$. The insets in the plots show the relaxation dynamics on a vertical log scale, clearly indicating extremely different relaxation times and the onset of the giant Mpemba effect. The fast and slow relaxation times can be obtained from the inverse of the slopes of the two curves and are given by $\tau_{f}≃3.1$ (fast time) and $\tau_{s}≃505$ (slow time). Note that, according to the perturbative analysis, $\tau_{f}$ is of order ∼1/g, where g≃0.4 is the spectral gap in the Liouvillian spectrum shown in (**a**), whereas $\tau_{f}/\tau_{s}≃0.006$ is of order $∼{\left(\epsilon /g\right)}^{2}=0.0125$. (**d**,**e**) Robustness of the giant Mpemba effect agains structural disorder in the lattices. Panel (**d**) shows a representative Liouvillian eigenvalue spectrum for a single realization of on-site potential disorder with strength W/J=0.5. Panel (**e**) depicts the disorder-averaged relaxation dynamics of states I and II, obtained from averaging over 20 disorder realizations. Other parameters are the same as in panels (**b**,**c**).

An illustrative example is shown in Figure 2. Here, networks A and B are modeled as tight-binding chains with $L_{A}$ and $L_{B}$ sites and uniform nearest-neighbor hopping rate *J*, coupled at their endpoints by a weak link of strength ϵ≪J [Figure 2a]. This model can be realized in a variety of experimental platforms, such as photonic quantum walks, superconducting qubit arrays, cold atoms in optical lattices, and trapped ions, to mention a few. For example, superconducting qubit arrays or resonator networks implement tight-binding chains with coherent hopping given by capacitive or inductive couplings, while local dephasing can be engineered via tunable noise channels or auxiliary dissipative elements; weak inter-chain links are achieved by adjusting edge couplings. Cold atoms in optical lattices provide another platform, where hopping is controlled by lattice depth, dephasing by laser-induced noise, and inter-chain barriers by local potential tuning. Finally, proposal to observe the Mpemba effect in photonic lattice models with controlled dephasing was given in [53]. Figure 2b shows the numerically computed eigenvalue spectrum of the Liouvillian L for parameter values ϵ/J=0.05, Γ/J=0.5, $L_{A}=10$ and $L_{B}=2$, clearly indicating the formation of the slow- and fast-mode manifolds, with N=1 slow modes and $\left(L^{2}-N\right)$ fast modes. The relaxation dynamics for states I and II are computed by numerically integrating Equation (15) using an accurate fourth-order variable-step Runge–Kutta method, and the trace distance $D(\rho \left(t\right),\rho_{E}^{\prime })$ from the equilibrium state is evaluated, as depicted in Figure 2c. The giant Mpemba effect is clearly visible: state I starts far from equilibrium yet equilibrates very rapidly, whereas state II begins closer to equilibrium but relaxes orders of magnitude more slowly. The effect is robust against static disorder in the system. As an illustrative example, we introduce on-site potential disorder in the coherent part of the Hamiltonian by adding the term $H\to H+\sum_{k=1}^{L}V_{k}\;a_{k}^{†}a_{k}$, where $V_{k}$ represents independent random variables uniformly distributed in the interval (−W/2,W/2). Figure 2d,e show the Liouvillian eigenvalue spectrum for a representative disorder realization [Figure 2d], together with the relaxation dynamics of states I and II averaged over 20 disorder realizations for a disorder strength of W/J=0.5. As one can see, even moderate-to-strong on-site disorder does not substantially modify the relaxation dynamics of the two states. This robustness originates from the presence of local dephasing, which suppresses phase coherence and therefore makes the slow-part of the Liouvillian spectrum largely insensitive to diagonal disorder in the underlying Hamiltonian.

**Figure 3:**
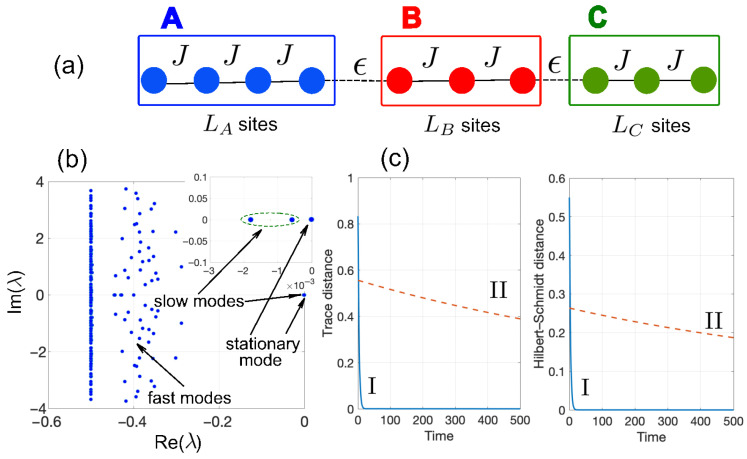
(**a**) Schematic of three dissipative bosonic chains *A*, *B* and *C* with nearest-neighbor hopping rate *J*, connected by two links with a weak coupling rate ϵ at the site edges. (**b**) Numerically computed eigenvalue spectrum λ of the Liouvillian L in the N=1 excitation sector for parameter values $L_{A}=8$, $L_{B}=6$, $L_{C}=4$, J=1, ϵ/J=0.05 and Γ/J=0.5. The inset in the figure depicts an enlargement of the spectrum near λ=0, clearly showing the zero eigenvalue, corresponding to the stationary state $\rho_{E}^{\prime }$, and the eigenvalues of the two slow modes. (**c**) Numerically computed behavior of the trace distance (left panel) and Hilbert–Schmidt distance (right panel) between ρ(t) and $\rho_{E}^{\prime }$ for two distinct initial states I and II. State I is defined by the initial condition $\rho \left(0\right)=p_{A}\left|1\rangle \langle 1\right|+p_{B}|L_{A}+L_{B}\rangle \langle L_{A}+L_{B}|+p_{C}\left|L\rangle \langle L\right|$, with $p_{A}=L_{A}/L=8/18$, $p_{B}=L_{B}/L=6/18$, $p_{C}=4/18$ and $L=L_{A}+L_{B}+L_{C}=18$. State II is defined by the initial condition $\rho \left(0\right)=\left(1/L_{A}\right)\sum_{k=1}^{L_{A}}\left|k\rangle \langle k\right|$.

While in the previous analysis we focused on M=2 weakly coupled subsystems, the emergence of the giant Mpemba effect can be observed when considering more than two subsystems. As an example, we can consider three tight-binding chains *A*, *B* and *C*, connected at the ends in series by a weak coupling link (hopping rate ϵ), as shown in Figure 3a. We assume an initial state

$$\rho \left(0\right)=p_{A}\rho_{A}\left(0\right)⊗{|0\rangle }_{B}\langle 0|⊗{|0\rangle }_{C}\langle 0|+p_{B}{|0\rangle }_{A}\langle 0|⊗\rho_{B}\left(0\right)⊗{|0\rangle }_{C}\langle 0|+$$

$$+p_{C}{|0\rangle }_{A}\langle 0|⊗{|0\rangle }_{B}\langle 0|⊗\rho_{C}\left(0\right),$$

where $\rho_{A}\left(0\right)$, $\rho_{B}\left(0\right)$ and $\rho_{C}\left(0\right)$ are arbitrary initial states in the *N*-particle excitation sector of *A*, *B* and *C* subsystems, and $p_{A}+p_{B}+p_{C}=1$. The number of slow modes is given by

$$N_{slow}=\left(\begin{array}{c} M+N-1\\ M-1 \end{array}\right)-1,$$

which is the combinatorial number of all possible distributions of *N* bosons in the *M* subsystems, minus one (corresponding to the stationary state). In the N=1 particle sector and for M=3, there are $N_{slow}=2$ slow modes, and the initial state ρ(0) does not excite slow modes whenever

(19) $$p_{A}=\frac{L_{A}}{L}\;,\;\;p_{B}=\frac{L_{B}}{L}\;,\;\;p_{C}=\frac{L_{C}}{L}$$

where $L_{A}$, $L_{B}$ and $L_{C}$ are the number of lattice sites in the three subsystems, and $L=L_{A}+L_{B}+L_{C}$. An example of the giant Mpemba effect in the three-chain system is illustrated in Figure 3b,c.

Finally, it is worth mentioning that the above analysis can be extended to manifolds with many excitations (N>1). Let us consider two weakly coupled bosonic networks A and B with *N* bosons. The bosonic *N*-particle subspace is given by

$$H_{N}=\mathrm{span}\{\;|n_{1},\ldots ,n_{L}\rangle \;\left|\;\sum_{i=1}^{L}n_{i}=N\right\}$$

where $|n_{1},\ldots ,n_{L}\rangle \equiv |n\rangle$ is the Fock basis with $n_{l}$ bosons at lattice site *l* (l=1,2,…,L) and $L=L_{A}+L_{B}$ is the total number of lattice sites. The dimension of $H_{N}$ is finite and given by

dimHN=N+L−1N.

When subsystems A and B are coupled via $\epsilon H_{I}$, the stationary state reads

$$\rho_{E}^{\prime }=\frac{1}{\dim H_{N}}\sum_{n=1}^{\dim H_{N}}|n\rangle \langle n|.$$

Its unicity is ensured by rather general criteria for uniqueness of stationary states in finite-dimensional space. In particular, according to the criterium introduced in Ref. [102], it is enough to prove that the operator algebra generated by $H=\sum_{i,j=1}^{L}h_{i,j}c_{i}^{†}c_{k}$ and the jump operators $L_{k}=c_{k}^{†}c_{k}$ is irreducible on $H_{N}$. The proof is basically the same as in the example in Section IV.D from Ref. [102], considering bosonic rather than fermionic particles, and requires a connected network. For a large excitation number *N*, the full density matrix becomes extremely large, with size $\dim H_{N}\times \dim H_{N}$, making numerical analysis increasingly challenging. However, to detect the onset of the giant Mpemba effect, rather than focusing on the relaxation of the full density matrix, one can instead analyze the relaxation dynamics of some good observables [48], such as the *two-point correlations*

(20) $$C_{n,m}\left(t\right)=\langle c_{n}^{†}c_{m}\rangle =\mathrm{Tr}\;\left(\rho \left(t\right)\;c_{n}^{†}c_{m}\right).$$

The study of two-point correlation functions is a standard and widely used approach to characterize the dynamics and relaxation of many-body systems, as they capture essential information about coherence, transport, and equilibration while remaining computationally tractable [103,104,105,106,107,108,109,110]. This reduction is also experimentally motivated: while full state tomography is typically infeasible in many-body systems due to the exponential growth of the Hilbert space, measurements of two-point correlations are routinely accessible across a broad range of quantum platforms. For quadratic Hamiltonians and local dephasing dissipators, the two-point correlations obey a closed set of linear Equations [106,107,108,109,110], namely one has

(21) $$\frac{d}{dt}C_{n,m}=-i\sum_{k}\left(h_{m,k}C_{n,k}-h_{k,n}C_{k,m}\right)-\Gamma \left(1-\delta_{n,m}\right)C_{n,m}.$$

Interestingly, the evolution equations for $C_{n,m}\left(t\right)$ are closed related to those of the density-matrix elements in the single-excitation (N=1) sector, i.e., to Equation (15): in fact, Equation (21) reduces to Equation (15) after formally letting $C_{n,m}\left(t\right)=\rho_{m,n}\left(t\right)$. Thus, even in the presence of multiple excitations, the relaxation of two-point correlations is governed by an effective Liouvillian equivalent to that of a single particle, indicating that the giant Mpemba effect can also be observed in a many-particle setting.

## 4. Conclusions and Discussion

We have demonstrated a general mechanism for realizing giant Mpemba effects in open quantum systems, where relaxation can be either hyper-accelerated or dramatically slowed depending on the initial state. The effect arises from the interplay of weak coherent interactions, dissipation, and the spectral structure of non-interacting stationary states. Specifically, we considered weakly coupled, particle-number-conserving bosonic networks, each of which independently relaxes rapidly to a unique stationary state. When a weak coherent interaction is introduced, the composite system generally develops slow metastable modes, leading to a hierarchy of relaxation timescales. By tailoring the interaction Hamiltonian $H_{I}$ such that certain initial states are effectively decoupled from these slow modes, one can achieve ultrafast relaxation even for states that are far from equilibrium. Conversely, other initial states—even those initially close to the stationary state—may remain trapped in the metastable manifold and decay anomalously slowly. The physical picture behind this mechanism is especially intuitive when considering coupled subsystems with very different Liouvillian spectral structures, since the larger subsystem typically dominates the relaxation dynamics. If the initial state has no overlap with the slow Liouvillian manifold, the system relaxes entirely on the fast timescale, independent of its nonequilibrium character, i.e., even when the large subsystem is very far from its equilibrium. Conversely, small overlaps with the slow manifold lead to extremely long relaxation times, even when the large subsystem is initially very close to its local stationary state. This asymmetry underlies the giant Mpemba effect observed in our model.

Beyond the specific model considered here, our framework is broadly applicable and suggests several promising extensions in both quantum and classical non-equilibrium systems. For example, the method could be applied to particle-conserving fermionic networks as well, where the Liouvillian spectrum and stationary states can be efficiently characterized via correlation matrices and no substantial distinctions arise between fermionic and bosonic systems in the relaxation dynamics of the two-point correlations [109,110]. Further, when the weak interaction is realized in the incoherent (dissipative) dynamics and $H_{A}=H_{B}=0$, the dynamics become fully dissipative and thus entirely classical, indicating that the framework can be applied to observe giant Mpemba effects in non-equilibrium classical models as well. Our results also connect naturally to recent developments in non-normal dynamics, exceptional points, and dynamical phase transitions in open quantum systems. The giant Mpemba effect represents an extreme sensitivity of relaxation times to initial conditions and microscopic couplings, a kind of dynamical analogue of critical phenomena in Liouvillian spectra. Exploring these connections further may provide new tools for controlling relaxation in complex quantum devices. Finally, while we focused on particle-number-conserving dynamics, it would be interesting to extend the same ideas to models without strict conservation laws.

In summary, the mechanism we have identified provides a flexible and robust way to engineer giant Mpemba effects in open quantum systems. Beyond their fundamental interest, such effects could find applications in quantum state preparation, thermalization control, metrology, and reservoir engineering.

## Data Availability

No data were generated or analyzed in the presented research.

## Appendix A. Slow-Relaxation Dynamics: Perturbative Analysis

We consider two dissipative subsystems *A* and *B*, whose uncoupled Liouvillian is

(A1) $$L_{0}=L_{A}⊗I_{B}+I_{A}⊗L_{B},$$

with $\left[L_{A},N_{A}\right]=\left[L_{B},N_{B}\right]=0$. We work in a fixed total particle-number sector *N*, consistent with the class of initial states considered in Equation (4) and with the fact that the interaction Hamiltonian $\epsilon H_{I}$ conserves the total number of excitations. For each integer 0≤n≤N, the unique steady states of $L_{A}$ and $L_{B}$ within their respective *n*-particle sectors are denoted by $\rho_{A}^{\left(n\right)}$ and $\rho_{B}^{\left(N-n\right)}$: $L_{A}\rho_{A}^{\left(n\right)}=0$ and $L_{B}\rho_{B}^{\left(N-n\right)}=0$. We assume rapid intra-sector relaxation: any state with a fixed local particle number relaxes to these steady states on a fast timescale $\tau_{f}∼1/g$, where *g* is the characteristic Liouvillian spectral gap of isolated subsystems. We note that, in the uncoupled limit (ϵ/g)=0, within a fixed total *N*-particle-number manifold, the stationary state of the full system, i.e., of the unperturbed Liouvillian $L_{0}$, is degenerate, as any state $\rho_{A}^{\left(n\right)}⊗\rho_{B}^{\left(N-n\right)}$ (n=0,1,2,…,N) is an eigenstate of $L_{0}$ with a zero eigenvalue. We now include a weak coherent interaction that preserves the total particle number:

(A2) $$L=L_{0}+\epsilon V,\;V\left(\cdot \right)=-i\left[H_{I},\cdot \right],\;\left(\epsilon /g\right)\ll 1,$$

which lifts the degeneracy of the stationary state leading to a unique stationary state $\rho_{E}^{\prime }$ for L. The other stationary states of the unperturbed Liouvillian $L_{0}$ will be mixed by the perturbation and will acquire small non-vanishing decay rates, corresponding to metastable states of the interacting system. The slow dynamics induced by $\epsilon H_{I}$ occur on the manifold

(A3) $$S=\mathrm{span}{\left\{\rho_{A}^{\left(n\right)}⊗\rho_{B}^{\left(N-n\right)}\right\}}_{n=0}^{N}$$

of the degenerate stationary states of $L_{0}$. Define the projector P onto S by

(A4) $$PX=\sum_{n=0}^{N}\mathrm{Tr}\left(\Pi^{\left(n\right)}X\right)\;\rho_{A}^{\left(n\right)}⊗\rho_{B}^{\left(N-n\right)},$$

where $\Pi^{\left(n\right)}$ projects onto states with *n* particles in *A* and (N−n) in *B*. The slow variables are the populations

(A5) $$p_{n}\left(t\right)=\mathrm{Tr}\left(\Pi^{\left(n\right)}\rho \left(t\right)\right),\;P\rho \left(t\right)=\sum_{n}p_{n}\left(t\right)\;\rho_{A}^{\left(n\right)}⊗\rho_{B}^{\left(N-n\right)},$$

and we denote Q=1−P.

The full master equation $\dot{\rho }=L_{0}\rho +\epsilon V\rho$ leads to the exact Nakajima–Zwanzig system (see, for instance, Ref. [111])

(A6) $$\frac{d}{dt}\left(P\rho \right)=\epsilon \;PVP\rho +\epsilon \;PVQ\rho ,$$

(A7) $$\frac{d}{dt}\left(Q\rho \right)=L_{0}Q\rho +\epsilon \;QVP\rho +\epsilon \;QVQ\rho .$$

In deriving the above equations, we used the property that $L_{0}$ annihilates each product equilibrium, i.e. $PL_{0}P=0$, $QL_{0}P=0$, $PL_{0}Q=0$, and $QL_{0}Q=L_{0}Q$. We assume that $L_{0}$ has a spectral gap g>0 on the Q-subspace, meaning that all nonzero eigenvalues satisfy Re(λ)≤−g. Consequently, the Q sector—i.e., Qρ(t)—relaxes on the fast timescale $\tau_{f}∼1/g$ (see Equation (A7)). In contrast, the perturbative assumption ϵ≪g ensures a clear separation of timescales: the evolution of Pρ(t) occurs on a much slower timescale $\tau_{s}$. Indeed, from Equation (A6), the derivative d(Pρ)/dt cannot change on a timescale shorter than ∼1/ϵ, establishing $\tau_{s}\gg \tau_{f}$.

To obtain the slow dynamics of Pρ(t), we start from the Nakajima–Zwanzig equation for the fast Q-component,

(A8) $$\frac{d}{dt}\left(Q\rho \right)=L_{0}Q\rho +\epsilon \;QVP\rho +O\left(\epsilon^{2}\right),$$

where the $O(\epsilon^{2})$ term ϵQVQρ has been neglected. Taking into account that $QL_{0}Q=L_{0}Q$, formally integrating and using that $e^{L_{0}t}$ decays on the fast timescale $\tau_{f}∼1/g$, we obtain

(A9) $$Q\rho \left(t\right)=\epsilon \int_{-\infty }^{t}ds\;e^{L_{0}\left(t-s\right)}QVP\rho \left(s\right)+O\left(\epsilon^{2}\right).$$

Since Pρ(t) varies slowly compared to the fast decay of $e^{L_{0}\left(t-s\right)}$, we may replace Pρ(s)→Pρ(t) (Markov approximation). Changing variables u=t−s and extending the upper limit to infinity yields

(A10) $$Q\rho \left(t\right)=\epsilon \int_{0}^{\infty }ds\;e^{L_{0}s}\;QVP\rho \left(t\right)+O\left(\epsilon^{2}\right),$$

which expresses the fast component as a perturbative functional of the slow variables. Hence for $t≳\tau_{f}$ we may write

(A11) $$\rho \left(t\right)=P\rho \left(t\right)+\epsilon \;\xi \left(t\right),\xi \left(t\right)=\int_{0}^{\infty }ds\;e^{L_{0}s}\;QVP\rho \left(t\right)\;+\;O\left(\epsilon \right),$$

showing explicitly that the dominant part of the state lies in the slow manifold S. Substituting the above expression for Qρ(t) into the P-equation gives, to second order in ϵ,

(A12) $$\begin{array}{ccc} \frac{d}{dt}P\rho \left(t\right) & = & \epsilon \;PVP\rho (t)\\ & + & \epsilon^{2}\;\left(\int_{0}^{\infty }ds\;PV\;e^{L_{0}s}\;QVP\right)\rho \left(t\right)+O\left(\epsilon^{3}\right) \end{array}$$

Applying $X\mapsto \mathrm{Tr}(\Pi^{\left(n\right)}X)$ finally yields the population dynamics:

(A13) $${\dot{p}}_{n}\left(t\right)=\epsilon \sum_{j}\alpha_{nj}p_{j}\left(t\right)+\epsilon^{2}\sum_{j}R_{nj}p_{j}\left(t\right)+O\left(\epsilon^{3}\right),$$

where

(A14) $$\alpha_{nj}=\mathrm{Tr}\;\left(\Pi^{\left(n\right)}V\left[\rho_{A}^{\left(j\right)}\;⊗\;\rho_{B}^{\left(N-j\right)}\right]\right),$$

(A15) $$R_{nj}=\int_{0}^{\infty }ds\;\mathrm{Tr}\;\left[\Pi^{\left(n\right)}V\;e^{L_{0}s}\;QV\left(\rho_{A}^{\left(j\right)}\;⊗\;\rho_{B}^{\left(N-j\right)}\right)\right].$$

The first-order term $\alpha_{nj}$ typically vanishes. Indeed, since V is a commutator,

$$\alpha_{nj}=-i\;\mathrm{Tr}\;\left(\left[\Pi^{\left(n\right)},H_{I}\right]\;\rho_{A}^{\left(j\right)}\;⊗\;\rho_{B}^{\left(N-j\right)}\right),$$

and whenever $H_{I}$ preserves each number sector (e.g. simple tunnelling between *A* and *B*) one has $\alpha_{nj}=0$. In this common situation the leading slow kinetics are $O(\epsilon^{2})$:

(A16) $${\dot{p}}_{n}\left(t\right)=\epsilon^{2}\sum_{j}R_{nj}p_{j}\left(t\right)+O\left(\epsilon^{3}\right).$$

Because $\sum_{n}\Pi^{\left(n\right)}=I$ and the trace of a commutator vanishes, one finds $\sum_{n}R_{nj}=0$, so that probability $\sum_{n}p_{n}\left(t\right)$ is conserved. Thus, $R_{nj}$ defines a classical rate matrix (under standard secular conditions), with one zero eigenvalue corresponding to the stationary populations of the interacting steady state and *N* nonzero eigenvalues of order $\epsilon^{2}$ describing the slow decay modes of the Liouvillian L.
